# The Condensate Wave Function of a Trapped Atomic Gas

**DOI:** 10.6028/jres.101.053

**Published:** 1996

**Authors:** F. Dalfovo, L. Pitaevskii, S. Stringari

**Affiliations:** Dipartimento di Fisica, Università di Trento, and Istituto Nazionale di Fisica della Materia, I-38050 Povo, Italy; Department of Physics, TECHNION, Haifa 32000, Israel; Kapitza Institute for Physical Problems, ul. Kosygina 2, 117334 Moscow; Dipartimento di Fisica, Università di Trento, and Istituto Nazionale di Fisica della Materia, I-38050 Povo, Italy

**Keywords:** classical turning point, Gross-Pitaevskii equation, kinetic energy, order parameter, quantized vorticity, Thomas-Fermi approximation

## Abstract

We discuss various properties of the ground state of a Bose-condensed dilute gas confined by an external potential. We devote particular attention to the role played by the interaction in determining the kinetic energy of the system and the aspect ratio of the velocity distribution. The structure of the wave function near the classical turning point is discussed and the drawback of the Thomas-Fermi approximation is explicitly pointed out. We consider also states with quantized vorticity and calculate the critical angular velocity for the production of vortices. The presence of vortex states is found to increases the stability of the condensate in the case of attractive interactions.

## 1. Introduction

In this paper we discuss some relevant properties of the ground state of a Bose-condensed atomic gas confined by an external potential. Our starting point is the Gross-Pitaevskii equation which gives the proper Schrödinger equation for the order parameter of an inhomogeneous dilute Bose-condensed gas at zero temperature. Using this equation it is possible to discuss various ground state properties of the system: the form of the atomic cloud, the role of the interatomic potential, the velocity distribution, and so on. All these quantities are essential for the interpretation of the recent experiments on Bose-Einstein condensation in ultracold alkali atom gases [1–3]. An important feature is the profound difference between systems interacting with repulsive and attractive forces. In the latter case, in particular, the stationary solution given by the Gross-Pitaevskii equation is of metastable type. If the number of atoms is too large such a solution becomes unstable and the system collapses. In this paper we will also discuss some rotational properties of the system, in particular the structure of vortices and the critical angular frequency needed to generate a rotational instability.

The Gross-Pitaevskii equation for the order parameter $\psi (r)=\langle \hat{\psi }(r)\rangle$ has the well known form [4]:

$$\left[-\frac{\hbar^{2}}{2m}\nabla^{2}+V_{\mathrm{ext}}(r)+\frac{4\pi \hbar^{2}a}{m}{\left|\psi (r)\right|}^{2}\right]\psi (r)=\mu \psi (r),$$ (1)

where *V*_ext_ is the external confining potential, which is usually chosen in the form of an anisotropic harmonic well. The role of interactions is accounted for by the non-linear term and is parametrized by the *s*-wave scattering length *a*. The quantity *μ* is the chemical potential and is fixed by imposing the proper normalization, *N* = ∫ *ρ* d***r***, to the density of the system *ρ* = |ψ|^2^. The Gross-Pitaevskii equation ignores interaction effects due to the atoms outside the condensate. This is an excellent approximation for a dilute Bose gas at low temperature where the depletion of the condensate is negligible.

An important question to discuss concerning the ground state of a trapped Bose gas is the role of the interatomic potential. At first sight one would in fact expect that the role of interactions be negligible in a dilute system, where the usual expansion parameter *a*^3^*ρ* is extremely small. Actually it turns out that the interaction can have a deep influence on the solution of Eq. (1), where its effect turns out to be fixed by the adimensional parameter *Na*/*a*_HO_, where $a_{\mathrm{HO}}=\sqrt{\hbar /(m\omega_{\mathrm{HO}})}$ is the harmonic oscillator length. This parameter can be indeed rather large despite the smallness of *a*^3^*ρ*. The final result is that the system is still fully Bose condensed, but the structure of its wave function can be strongly affected by the interatomic forces.

For the above reasons it is useful to discuss the solution of the Gross-Pitaevskii equation in two relevant limits: the noninteracting model and the strongly repulsive limit, which corresponds to the Thomas-Fermi approximation. We will make a comparison with the exact numerical solution of the Gross-Pitaevskii equation in order to point out the role of the interaction. We will devote special attention to the structure of the condensate wave function near the boundary, close to the classical turning point, and we will finally study the case of quantized vortices.

## 2. The Noninteracting Model

When the scattering length *a* vanishes, the problem reduces to the solution of a one-body Schrödinger equation. Let us put *a* = 0 in Eq. (1) and take the external potential as an anisotropic harmonic oscillator:

$$V_{\mathrm{ext}}(r)=\frac{m}{2}\;(\omega_{⊥}^{2}\;x^{2}+\omega_{⊥}^{2}\;y^{2}+\omega_{z}^{2}\;z^{2}).$$ (2)

The ground state wave function becomes

$$\psi (r)=\sqrt{\frac{N}{a_{⊥}^{3}}}\lambda^{1/4}\pi^{-3/4}\exp\left[-\frac{1}{2a_{⊥}^{2}}\;(x^{2}+y^{2}+\lambda z^{2})\right],$$ (3)

where *λ* = *ω_z_*/*ω*_⊥_. The Gaussian has different transverse and vertical widths. In particular one has $\langle x^{2}\rangle =\langle y^{2}\rangle =(1/2)a_{⊥}^{2}$ and $\langle z^{2}\rangle =(1/2)\lambda^{-1}a_{⊥}^{2}$. The chemical potential is (1 + *λ*/2) *ħω*_⊥_ and coincides with the energy per particle, while the kinetic energy per particle has the simple form

$$\frac{E_{\mathrm{kin}}}{N}=\frac{1}{2m}\langle p^{2}\rangle =\frac{1}{2}(1+\lambda /2)\hbar \omega_{⊥}.$$ (4)

An interesting quantity to discuss is the ratio $\sqrt{\langle p_{z}^{2}\rangle /\langle p_{x}^{2}\rangle }$ which provides a measure of the *aspect ratio*, characterizing the anisotropy of the velocity distribution. Using the wave function Eq. (3) one finds

$$\sqrt{\langle p_{z}^{2}\rangle /\langle p_{x}^{2}\rangle }=\sqrt{\langle x^{2}\rangle /\langle z^{2}\rangle }=\sqrt{\lambda }.$$ (5)

Values of the aspect ratio different from 1 reflect a peculiar and unique feature of Bose-Einstein condensation.

## 3. The Strongly Repulsive Limit (Thomas-Fermi Approximation)

The opposite limit is obtained when the interaction is so strong, or the number of particles so large, that the kinetic energy term Δ^2^ψ can be neglected in the Gross-Pitaevskii equation, Eq. (1). It corresponds to very large values of the dimensionless parameter

$$u=\frac{8\pi aN}{a_{⊥}}.$$ (6)

Also in this case the solution of the Gross-Pitaevskii equation is trivial and the wave function has the form:

$$\rho \left(r\right)={\left|\psi \left(r\right)\right|}^{2}=\frac{m}{4\pi \hbar^{2}a}\;\left[\mu -V_{\mathrm{ext}}\left(r\right)\right]$$ (7)

if the right hand side is positive, and *ρ* = 0 elsewhere. The chemical potential is easily calculated by imposing the normalization condition ∫ *ρ* d***r*** = *N*. One finds

$$\mu =\frac{1}{2}{\left(\frac{15}{8\pi }\;\lambda u\right)}^{2/5}\hbar \omega_{⊥}.$$ (8)

Due to the different scaling properties of the wave function with respect to the variable *z* (compare Eqs. (3) and (7)), the aspect ratio $\sqrt{\langle p_{z}^{2}\rangle /\langle p_{x}^{2}\rangle }$ in this case is equal to *λ* differently from the noninteracting case Eq. (5).

The wave function Eq. (7) is expected to approximate well the exact solution of the Gross-Pitaevskii equation Eq. (1) for large *N*, apart from the structure of the surface region where the exact wave function has to vanish smoothly. Some relevant observables, as the kinetic energy, can be significantly affected by this surface structure, as we will see in Sec. 5.

## 4. Solution of the Gross-Pitaevskii Equation

The Gross-Pitaevskii equation, Eq. (1) can be solved numerically [5,6]. We transform the differential equation in a functional minimization and use a steepest descent method to solve the minimization problem on a grid of points. As an example of atoms with repulsive interaction we choose ^87^Rb, as in the experiment of Ref. [1]. For the *s*-wave triplet-spin scattering length we use *a* = 100*a*_0_, where *a*_0_ is the Bohr radius. The asymmetry parameter is taken $\lambda =\omega_{z}/\omega_{⊥}=\sqrt{8}$ and the axial frequency *ω_z_*/2*ħ* = 220 Hz. The corresponding characteristic length is *a*_'⊥_ = 1.222 × 10^−4^ cm.

**Fig. 1:**
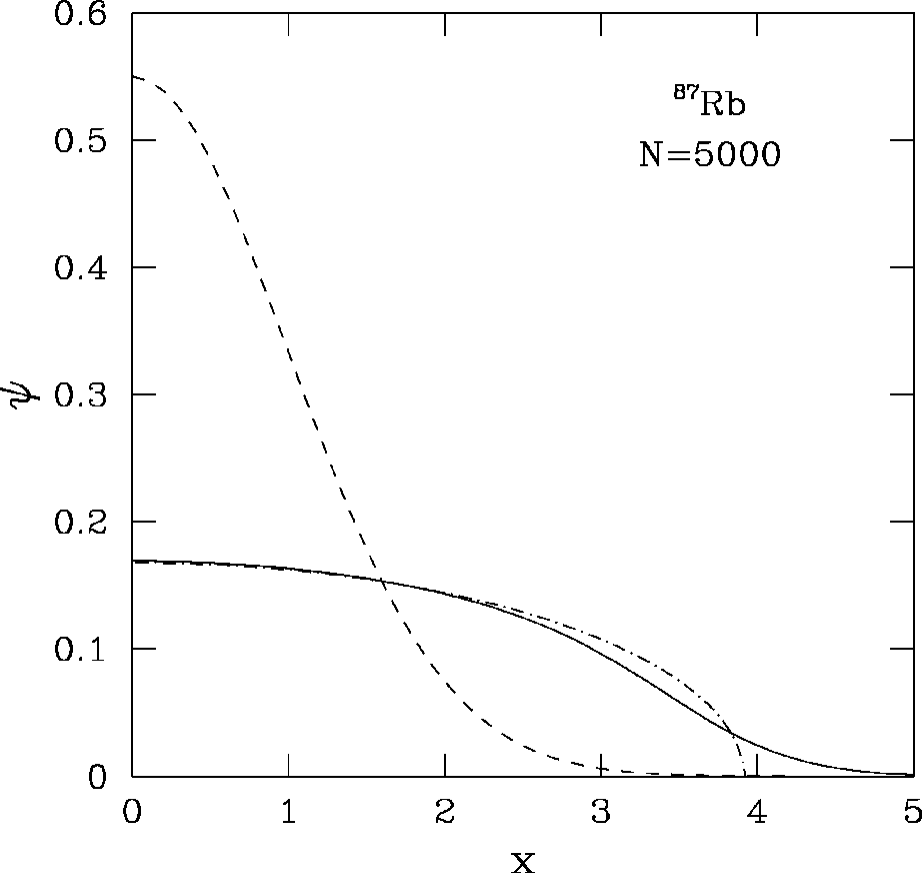
Ground state wave function (in arbitrary units) along *x* (in units *a*_⊥_) for 5000 atoms of ^87^Rb. Dashed line: noninteracting case. Dot-dashed line: Thomas-Fermi approximation. Solid line: numerical solution of the Gross-Pitaevskii equation.

Results for the chemical potential and the energy per particle are shown in Table 1. Both quantities are expressed in units of *ħω*_⊥_, or of the equivalent temperature *ħω*_⊥_/*k*_B_ = 3.73 nK. The partial contributions to the energy per particle coming from the kinetic energy (kin), the harmonic oscillator potential (HO) and the internal potential energy (pot) are also given. The *N* = 1 case coincides with the noninteracting anisotropic harmonic oscillator: in this case the total energy per particle coincides with the analytic value (1 + *λ*/2) = 2.414. When *N* increases the repulsion among atoms tends to lower the central density, expanding the cloud of atoms towards regions where the trapping potential is higher. A typical profile of the condensate wave function ψ is plotted along the *x*-axis for *N* = 5000 in Fig. 1. The exact minimization of the Gross-Pitaevskii functional (solid line) is compared with the noninteracting case (dashed line) and the Thomas-Fermi limit (dot-dashed).

When *N* is large we observe an increase of both interaction and harmonic oscillator potential energy per particle (the latter effect follows from the expansion of the cloud). Conversely, the kinetic energy per particle decreases because the density distribution is flattened. In the strongly repulsive limit, *N* → ∞, one should find that the internal potential energy is much greater than the kinetic energy. Indeed the convergence towards this limit turns out to be rather slow as we will show in the next section.

**Fig. 2:**
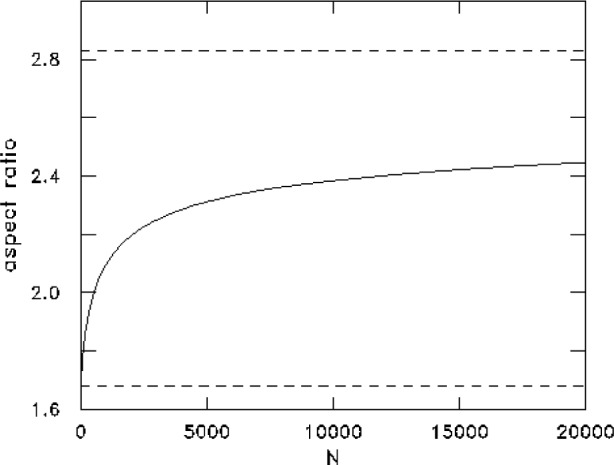
Ratio of the axial to transverse average velocity as a function of *N* in ^87^Rb. The lower and upper dashed lines corresponds to $\sqrt{\lambda }$ and *λ*, respectively.

Another interesting quantity which can be easily calculated from the ground state wave function is the aspect ratio of the velocity distribution, that is the ratio $\surd \langle p_{z}^{2}\rangle /\langle p_{x}^{2}\rangle$. This quantity is equal to $\sqrt{\lambda }$ in the noninteracting case and should approach *λ* in the strongly repulsive limit. The numerical results, as a function of *N*, are shown in Fig. 2. The two limiting cases are shown as dashed lines. One clearly sees that the convergence to the value 2.828 = *λ* is very slow; the aspect ratio remains well below the asymptotic value even for *N* = 20000. The aspect ratio measured in Ref. [1] is estimated to be about 50 % larger than the noninteracting value, while the number of particles is of the order of 5000. The agreement with our results is good, even if one has to consider that the experimental estimate implicitly assumes a ballistic expansion of the atoms after switching off the external trap. The effects of the interaction on the expansion of the gas should be explicitly taken into account in order to draw more definitive conclusions.

As an example of atoms with attractive interaction we choose ^7^Li, as in the experiment of Ref. [2]. For the *s*-wave triplet-spin scattering length we use *a* = −27*a*_0_. The axial frequency reported in Ref. [2] is *ω_z_*/2*π* = 117 Hz and the corresponding characteristic length is *a*_⊥_ = 2.972 × 10^−4^ cm. The transverse frequency is *ω_z_*/2*π* = 163 Hz, so that the asymmetry parameter is *λ* = *ω_z_/ω*_⊥_ = 0.72.

The first important point to stress is that Gross-Pitaevskii functional has no global minimum for negative scattering length. This reflects the tendency of the system to collapse. For spatially inhomogeneous systems, however, the zero-point energy can exceed the attractive potential, producing local minima of the functional when the density of atoms is not too high.

**Fig. 3:**
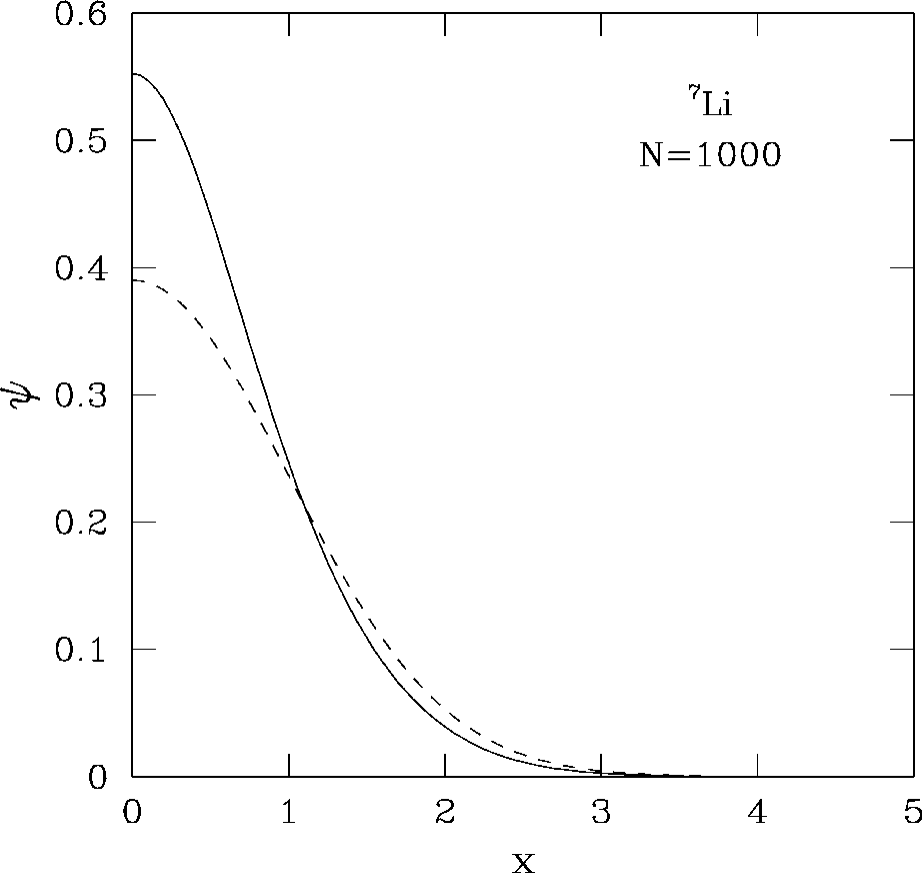
Ground state wave function (in arbitrary units) along *x* (in units *a*_⊥_) for 1000 atoms of ^7^Li. Dashed line: noninteracting case. Solid line: numerical solution of the Gross-Pitaevskii equation.

The most striking difference with respect to the repulsive case is that here the central density of the cloud increases rapidly with *N*, as shown in Fig. 3. This is the effect of adding more and more attractive potential energy. When the central density reaches a certain critical limit the system collapses and the solution of the Gross-Pitaevskii equation does not converge anymore. In ^7^Li, with the input parameters given above, the critical number *N* turns out to be about 1400. In [6] we have found that a stationary solution of the Gross-Pitaevskii equation with larger values of *N* can be obtained if a vortex line is present in the system. The possible occurrence of vortices in these trapped Bose gases will be discussed in the Sec. 6.

## 5. Wave Function at the Boundary

As one clearly sees in Fig. 1, the Thomas-Fermi approximation fails to reproduce the structure of the order parameter at the surface of the atomic cloud in the case of positive scattering length (repulsive interaction). Several measurable quantities can be significantly affected by the behavior of the wave function in this region. In order to provide a good model for these quantities one has to go beyond the Thomas-Fermi approximation. One of these relevant observables is the kinetic energy

$$E_{\mathrm{kin}}=\frac{\hbar^{2}}{2m}\int dr|\nabla \psi {|}^{2}.$$ (9)

In fact the Thomas-Fermi approximation (7) for the wave function is not appropriate to evaluate *E*_kin_; it produces a logarithmic divergence in the integrand of Eq. (9), occurring at the classical turning point, where *V*_ext_ = *μ*. This reveals that the evaluation of *E*_kin_ requires higher accuracy in the description of the boundary region. In order to provide the proper description of the condensate wave function near the boundary we have recently proposed [7] a suitable expansion of the Gross-Pitaevskii equation near the classical turning point. The resulting analysis allows one to obtain the proper expansion for the kinetic energy. We briefly sketch here the case of isotropic traps (*a*_⊥_ = *a_z_* = *a*_HO_).

Let *R* be the boundary of system spherical system, determined by the equation *μ* = *V*_ext_(*R*). Near this point one can carry out the expansion

$$V_{\mathrm{ext}}(r)-\mu ≃(r-R)F$$ (10)

where $F=m\omega_{\mathrm{HO}}^{2}R$ is the modulus of the attractive external force evaluated at *r* = *R*. Close to the boundary, where |*r* − *R*| ≪ *R*, the Gross-Pitaevskii equation takes the form

$$-\frac{\hbar^{2}}{2m}\frac{d^{2}}{dr^{2}}\psi +(r-R)F\psi +\frac{4\pi \hbar^{2}a}{2m}\psi^{3}=0.$$ (11)

Let us now introduce the dimensionless variable

$$\xi =\frac{\left(r-R\right)}{d}$$ (12)

where

$$d={\left(\frac{2m}{\hbar^{2}}F\right)}^{-1/3}$$ (13)

is a typical thickness of the boundary giving, as we will see later, the distance from the classical radius *R* where the Thomas-Fermi approximation starts failing. Then we introduce the dimensionless function *ϕ* defined by

$$\psi (r)=\frac{1}{d{\left(8\pi a\right)}^{1/2}}\;\phi (\xi ),$$ (14)

in terms of which the Gross-Pitaevskii equation, Eq. (11) takes the universal form

$$\phi^{\prime \prime }-(\xi +\phi^{2})\phi =0.$$ (15)

**Fig. 4:**
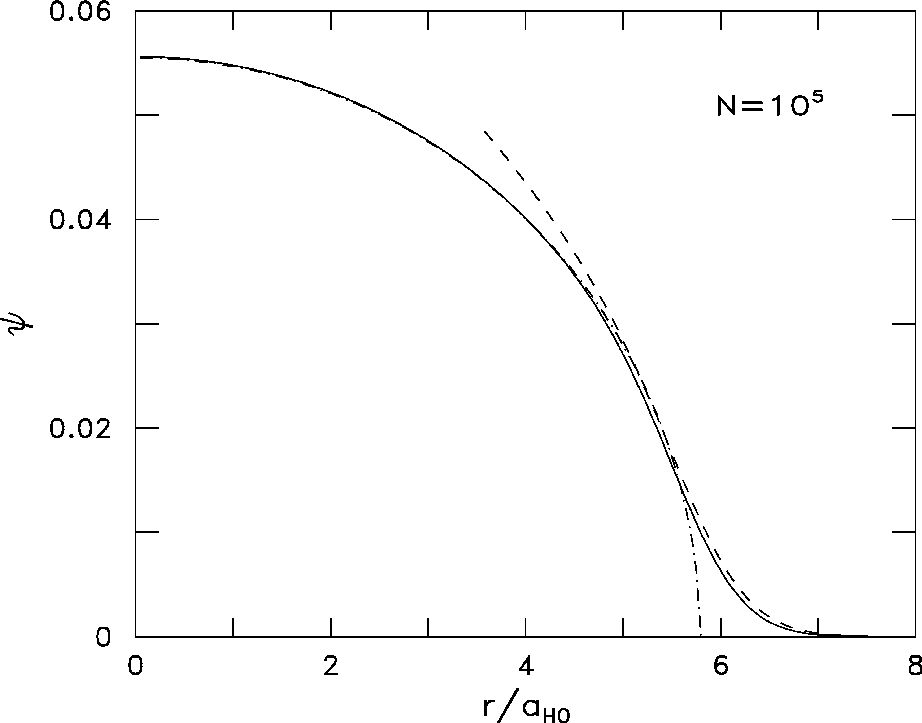
Condensate wave function (arbitrary units) for 10^5^ atoms of ^87^Rb in a spherical harmonic trap of length *a*_HO_. Solid line: numerical solution of the Gross-Pitaevskii equation, Eq. (1). Dot-dashed line: Thomas-Fermi approximation, Eq. (7) (indistinguishable from the solid line in the inner part). Dashed line: surface profile obtained from the universal equation, Eq. (15).

Its solution provides, via Eqs. (12–14), the proper structure of the condensate wave function near the classical turning point *R*. It is worth noting that Eq. (15) does not depend on the form of the external potential nor on the size of the interatomic force. These physical parameters enter the transformations Eqs. (12) and (14) which fix, together with the solution of Eq. (15), the actual behavior of the wave function ψ. Equation (15) can be solved numerically. The function *ϕ* behaves like $\sqrt{-\xi }$ for *ξ* → −∞ and like *ξ*^−1/4^ exp[− (2/3)*ξ*^3/2^] in the opposite limit *ξ* → ∞. The corresponding condensate wave function ψ matches the Thomas-Fermi wave function at the left of the classical turning point and follows closely the solution of the Gross-Pitaevskii equation in the external surface profile. An example is given in Fig. 4 for 10^5^ atoms of ^87^Rb in a spherical trap.

The kinetic energy can be calculated by matching properly the Thomas-Fermi approximation and the solution of the universal equation, Eq. (15). This yields the result

$$\frac{E_{\mathrm{kin}}}{N}=\frac{5}{2}\frac{\hbar^{2}}{mR^{2}}\;\left(\log\frac{R}{a_{\mathrm{HO}}}-0.259\right),$$ (16)

where the radius *R* is related to *N* by the equation

$$N=\frac{R^{5}}{15\;a\;a_{\mathrm{HO}}^{4}}.$$ (17)

**Fig. 5:**
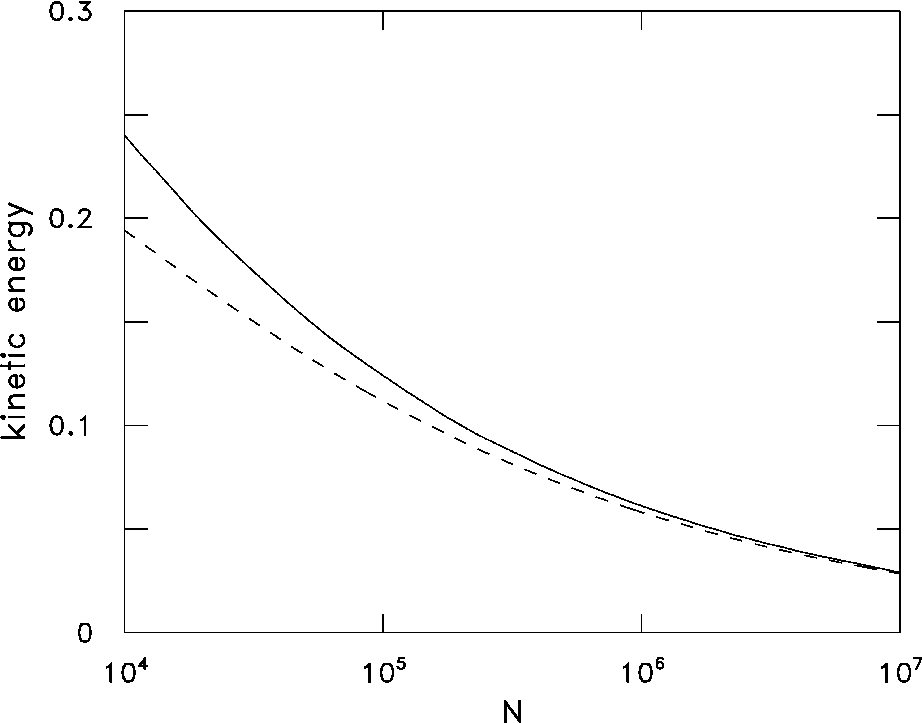
Kinetic energy per particle, in units *ħω*_HO_, for ^87^ Rb in a spherical harmonic trap as a function of the number of condensed atoms. Solid line: from the solution of the Gross-Pitaevskii equation (1). Dashed line: approximation Eq. (16).

Equation (16) provides the proper behavior of the kinetic energy in the large *N* limit where *R* ≫ *a*_HO_. In Fig. 5 we compare the results obtained from Eq. (16) and from the Gross-Pitaevskii equation. One can see that the convergence is reached for relatively large values of *N*.

## 6. Vortices

The structure of vortices in a trapped Bose gas can be naturally investigated in the present formalism. Let us consider states having a vortex line along the *z*-axis and all the atoms flowing around it with quantized circulation. One can write the axially symmetric condensate wave function in the form

Ψ(r)=ψ(r)exp[iS(r)] (18)

where $\psi (r)=\sqrt{\rho (r)}$ is the modulus, while the phase *S* acts as a velocity potential: *υ* = (*ħ*/*m*)∇*S*. By choosing *S* = *κϕ*, where *ϕ* is the angle around the *z*-axis and *κ* is an integer, one has vortex states with tangential velocity

$$\upsilon =\frac{\hbar }{mr_{⊥}}\;\kappa ,$$ (19)

with $r_{⊥}^{2}=x^{2}+y^{2}$. The number *κ* is the quantum of circulation, and the angular momentum along *z* is *Nκħ*.

If the complex wave function Eq. (18) is used in the derivation of the Gross-Pitaevkii equation, one gets

$$\left[-\frac{\hbar^{2}}{2m}\nabla^{2}+\frac{\hbar^{2}\kappa^{2}}{2mr_{⊥}^{2}}+\frac{m}{2}(\omega_{⊥}^{2}r_{⊥}^{2}+\omega_{z}^{2}z^{2})+\frac{4\pi \hbar^{2}a}{m}|\psi (r){|}^{2}\right]\;\psi (r)=\mu \psi (r),$$ (20)

which differs from Eq. (1) only for the addition of a centrifugal potential. This new term forces the solution ψ to vanish on the *z*-axis for *κ* ≠ 0.

For noninteracting particles one falls again in the case of the stationary Schrödinger equation for the anisotropic harmonic potential. For instance the *κ* = 1 solution has the form

$$\psi (r)\propto r_{⊥}\;\exp\;\left[-\frac{1}{2a_{⊥}^{2}}\;(r_{⊥}^{2}+\lambda z^{2})\right].$$ (21)

To get the energy per particle for the *κ* ≠ 0 states one has simply to sum *κħω*_⊥_ to the energy per particle of the ground state without vortices.

In the interacting case the kinetic energy can not be neglected even for large *N*, since it determines the structure of the vortex core. In particular, the balance between the kinetic energy and the interaction energy fixes a typical distance over which the condensate wave function can heal. For a dilute Bose gas the *healing length* is given by

$$\xi ={\left(8\pi \rho a\right)}^{-1/2}$$ (22)

where *ρ* is the density of the system. In the case of a vortex it corresponds to the distance over which the wave function increases from zero, on the vortex axis, to the bulk density. For the trapped atoms in the *N* → ∞ limit one finds

$$\frac{\xi }{R}={\left(\frac{a_{⊥}}{R}\right)}^{2}.$$ (23)

Thus the healing length is small compared with the size of the cloud if *R* is much bigger than *a*_⊥_.

The critical angular velocity required to produce vortex states is easily calculated once the energies of the states with and without vortices is known. One has to compare the energy of a vortex state in frame rotating with angular frequency *Ω*, that is (*E* − *ΩL_z_*), with the energy of the ground state with no vortices. Since the angular momentum per particle is *κħ*, the critical angular velocity is

$$\Omega_{c}={\left(\hbar \kappa \right)}^{-1}[{\left(E/N\right)}_{\kappa }-{\left(E/N\right)}_{0}].$$ (24)

In the noninteracting case the difference of energy per particle is simply *κħω*_⊥_, so that *Ω*_c_ = *ω*_⊥_.

**Fig. 6:**
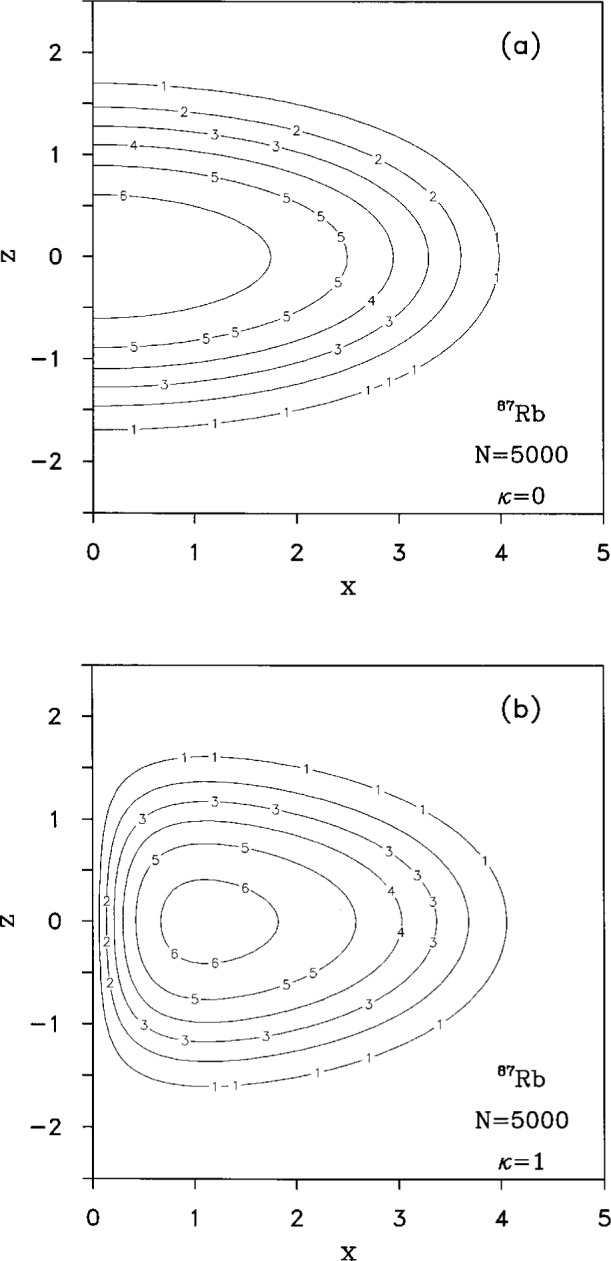
Wave function, in arbitrary units, of 5000 ^87^Rb atoms. Spatial coordinates in units *a*_⊥_. a) Ground state. b) Vortex state with *κ* = 1.

**Fig. 7:**
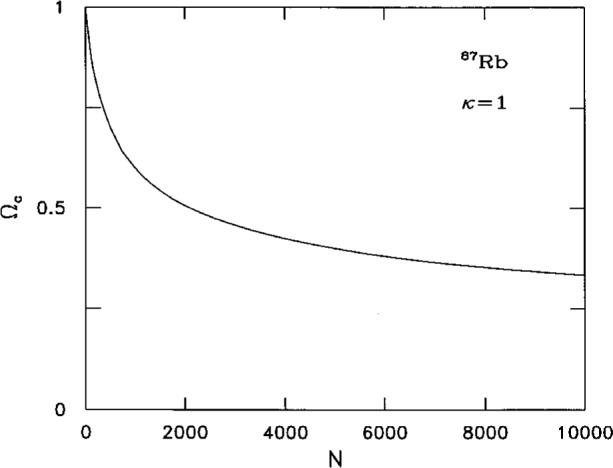
Critical angular velocity, in units *ω*_⊥_, for the formation of *κ* = 1 vortices in ^87^Rb vapor as a function of *N*.

We have solved numerically the Gross-Pitaevskii equation (20) both for rubidium and lithium. In Fig. 6 we show the wave function of a cloud of 5000 ^87^Rb atoms; the *κ* = 1 wave function (Fig. 6b), which corresponds to atoms flowing around the *z*-axis with angular momentum *Nħ*, is compared with the *κ* = 0 ground state (Fig. 6a). The atoms are pushed away from the axis forming a toroidal cloud. From the energy of the vortex states we calculate the critical angular velocity, through Eq. (24). The results for *κ* = 1 are shown in Fig. 7. The critical angular velocity decreases rapidly with *N*. For *N* > 5000 it is less than 40 % of the noninteracting value, given by the transverse angular frequency *ω*_⊥_ of the trap. The *healing length* is the distance over which the wave function grows from zero to the *bulk* value. In the limit of large systems it can be approximated by Eq. (22) with *ρ* equal to the density in the central part of the toroidal distribution. Both the estimate of *ξ* and *Ω*_c_ obtained in this way are in qualitative agreement with the behavior of the numerical solutions.

**Fig. 8:**
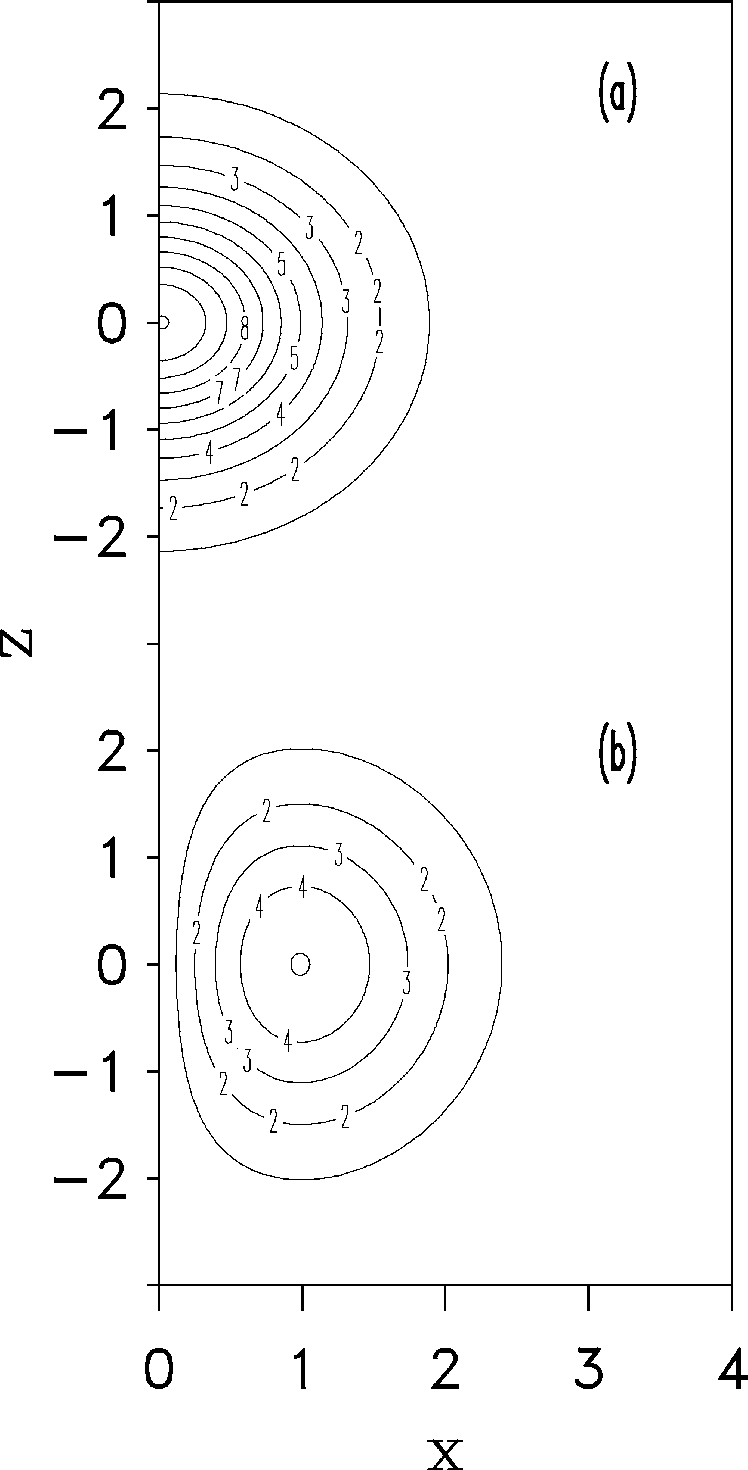
Wave function, in arbitrary units, of 1000 ^7^Li atoms. Spatial coordinates in units *a*_⊥_. a) Ground state. b) Vortex state with *κ* = 1.

Coming back to the question of the stability for negative scattering length, we notice that, when the local minimum associated with wave functions of the form shown in Fig. 3 disappears, nothing prevents *a priori* the existence of other local minima associated with different configurations. Such configurations should have local density lower than the critical one. A natural way to obtain a favourable situation is to move the atoms away from the *z*-axis, conserving the total number of particles. This happens in the presence of a vortex. In Fig. 8 we show the wave function for 1000 ^7^Li atoms with no vortices (Fig. 8a) and with an axial vortex of unit circulation (Fig. 8b). We use the same units in both cases, so one can see that the maximum value of the wave function inside the toroidal distribution of the vortex is approximately a factor two lower than the central value in the state with no vorticity (the density is four times smaller). The critical angular frequency for the formation of the vortex state in Fig. 8 is 1.12 times the transverse angular frequency of the trap. In systems with attractive interaction the critical angular velocity is larger than for noninteracting particles, while the opposite is true for repulsive interaction. This is because it costs internal potential energy to lower the average density, as the vortex does, for attractive interactions. However, once a vortex is created, the corresponding state is more stable than in the absence of vorticity: one can put more atoms inside the rotating cloud before reaching the critical density for the final collapse. Indeed we find local minima of the Gross-Pitaevskii functional for *N* much larger than 1400 if *κ* > 0. For *κ* = 1 we find a critical value of *N* ≃ 4000; for *κ* = 2 and 3 we find critical values of 6500 and 8300, respectively. It is worth mentioning that the number of particles in the condensate reported in the experimental work of Ref. [2] is an order of magnitude higher than the critical value for the stability of the Gross-Pitaevskii solution without vorticity (*N* ≃ 1400). The presence of vortices might explain the large size of the observed Bose-condensed gas. Further experimental data are needed to draw more definitive conclusions.

## Figures and Tables

**Table 1:** Results for the ground state of ^87^Rb atoms in a trap with *ω_z_*/2*π* = 220 Hz and $\lambda =\omega_{z}/\omega_{⊥}=\sqrt{8}$. Chemical potential and energy in units *ħω*_⊥_

*N*	*μ*	(*E*/*N*)	(*E*/*N*)_kin_	(*E*/*N*)_HO_	(*E*/*N*)_pot_
1	2.414	2.414	1.207	1.207	0.000
100	2.88	2.66	1.06	1.39	0.21
200	3.21	2.86	0.98	1.52	0.36
500	3.94	3.30	0.86	1.81	0.63
1000	4.77	3.84	0.76	2.15	0.93
2000	5.93	4.61	0.66	2.64	1.32
5000	8.14	6.12	0.54	3.57	2.02
10000	10.5	7.76	0.45	4.57	2.74
15000	12.2	8.98	0.41	5.31	3.26
20000	13.7	9.98	0.38	5.91	3.68
